# Tele-nursing awareness, needs, and related influences in T2DM patients: qualitative descriptive study

**DOI:** 10.1186/s12912-025-03048-2

**Published:** 2025-04-29

**Authors:** Jian Zhou, Xinxin Fan, Qiulin Xie, Hong Qi, Zongting Luo

**Affiliations:** 1 Department of Orthopedics, The First Affiliated Hospital of Chengdu Medical College, Chengdu, China; 2 School of Nursing, Chengdu Medical College, Chengdu, China; 3 Health Science Center, Yangtze University, Jingzhou, China; 4 Department of Nursing, The Third People’s Hospital of Chengdu, Chengdu, China; 5 No. 278, Middle Section, Baoguang Avenue, Xindu District, Chengdu City, Sichuan Province 610500 China

**Keywords:** Tele-nursing, Qualitative research, Awareness, Needs, T2DM

## Abstract

**Background:**

Tele-nursing, which utilizes digital health tools, has the potential to enhance the management of Type 2 Diabetes (T2DM). However, patients’ awareness, needs, and barriers to using these services are not well understood. This study employed qualitative interviews to explore the perceptions of patients with T2DM towards tele-nursing and to identify factors influencing their adoption, with the aim of providing tailored strategies for effective service implementation.

**Methods:**

A descriptive qualitative research design was used. From June to August 2023, a purposive sampling method was used to select 20 T2DM patients from a hospital, following the principle of maximum variation. Semi-structured interviews were conducted, and the data were analyzed using thematic analysis.

**Results:**

Four main themes were identified: insufficient awareness and willingness to use tele-nursing, the need for tele-nursing services, facilitators of tele-nursing, and barriers to tele-nursing.

**Conclusion:**

The awareness of tele-nursing among T2DM patients needs significant improvement. Patients expressed clear demands for tele-nursing services, including health education, dietary guidance, blood sugar monitoring, and medication reminders. However, the adoption of tele-nursing is influenced by factors such as service accessibility, scope, technological challenges, and associated costs. Tele-nursing should address the specialized nursing needs of T2DM patients by leveraging remote technology to provide personalized and flexible care options that align with their evolving requirements.

## Background

According to the International Diabetes Federation [1], there were approximately 465 million people worldwide with diabetes, and this number was expected to rise to 700 million by 2045. China currently has the highest number of T2DM patients globally making it one of the major public health issues [2]. The goals of T2DM prevention and treatment are to prevent complications, reduce disability and mortality rates, and improve patients’ quality of life through lifestyle modifications and appropriate use of glucose-lowering medications [3]. Hospitals play a key role in managing T2DM by providing treatment and addressing acute issues, while self-management, essential for daily blood glucose control, lifestyle adjustments, and emotional well-being, largely depends on community nursing support [4, 5]. Thus, enhancing self-management skills has become a core objective of community nursing for diabetes.

Historically, community nursing in China was unevenly developed, with insufficient human resources and incomplete functions in primary healthcare unit [6, 7]. Additionally, patients had limited awareness of community nursing, overly relying on large hospitals and neglecting the role of primary healthcare institutions [8, 9]. This resulted in low efficiency in enhancing the self-management abilities of diabetic patients in the community [10]. However, factors such as limited time, space, and human resources have led to inadequate access to medical and nursing services in these hospitals [11–13]. Telemedicine refers to the delivery of healthcare services and health management remotely using information and communication technologies, such as telephones, the internet, and video conferencing [14, 15]. It extended medical resources to communities and primary care settings through teleconsultations and remote guidance [16, 17], providing new opportunities for enhancing the self-management of diabetic patients. Tele-nursing, a branch of telemedicine, enables nurses to remotely assess patients’ health, deliver educational interventions, and provide care to patients in the comfort of their homes [18]. Studies indicated that tele-nursing had advantages over telemedicine in terms of cost investment and socioeconomic benefits to some extent.For example, a study by Advent Health University pointed out that the cost of tele-nursing visits is generally lower than in-person visits, reducing visitation expenses and increasing productivity by decreasing employee absence [15]. Additionally, an article in HealthTech Magazine mentioned that telehealth can improve follow-up appointments and enable more immediate connections between patients and healthcare providers, thereby reducing healthcare costs and improving patient health outcomes [19]. Tele-nursing is also more conducive to implementation in communities and can compensate for the lack of medical resources in these areas [20, 21]. It is important to acknowledge that tele-nursing cannot replace face-to-face nursing care. However, tele-nursing can overcome the limitations of time and space, playing an active role in health education, disease management, dissemination of health knowledge and skills, and promoting self-management [22]. At present, research on internet-based telecare mainly focuses on policy research and general nursing care, but there is still a significant lack of in-depth studies in specialized disease care [23].

To better facilitate the transition from general nursing to specialized care for diabetes, it is necessary to analyze patients’ awareness of tele-nursing and related factors from a subjective and objective perspective. Therefore, the study aimed to explore the awareness, needs, and influencing factors of tele-nursing among T2DM patients through descriptive qualitative research. This study would provide an intuitive perspective and viewpoint for tele-nursing in specialized care for T2DM, aiding in the self-management and improvement of quality of life for T2DM patients.

## Materials and methods

### Research design

A descriptive and exploratory qualitative research design was employed to conduct semi-structured interviews with T2DM patients, aiming to explore their awareness and needs regarding tele-nursing services. Tthematic analysis, following the framework of Braun and Clarke [24], was used to systematically analyze the data, and identify key themes related to patient experiences. The reporting of this study adhered to the Consolidated Criteria for Reporting Qualitative Research (COREQ) [25], ensuring transparency and rigor in both the data collection and analysis processes.

### Study participants

Between June and August 2023, a purposive sampling strategy was implemented to recruit T2DM patients following maximum variation criteria [26]. Participants were selected with consideration of demographic and socioeconomic factors, including age, gender, educational attainment, household monthly income, residential area, and medical insurance type. This sampling approach aimed to ensure comprehensive representation of tele-nursing service awareness and needs across diverse patient populations. Twenty T2DM patients from a tertiary hospital in Chengdu, were selected. Inclusion criteria: (1) Met the diagnostic criteria of the ‘China Type 2 Diabetes Prevention and Control Guidelines (2021 Edition)’ [27]; (2) Patients aged ≥ 18 years and able to communicate; (3) Agreed to participate and signed informed consent.Exclusion criteria: (1) Cognitive impairments (e.g., Alzheimer’s, severe post-stroke sequelae) that hinder understanding and communication; (2) Severe mental illnesses (e.g., schizophrenia, bipolar disorder) affecting emotional stability; (3) Advanced cancer, especially during chemotherapy or radiotherapy; (4) Severe cardiovascular diseases (e.g., heart failure, recent myocardial infarction) limiting participation; (5) End-stage renal or liver disease (e.g., dialysis patients) impairing physical ability; (6) Severe respiratory diseases (e.g., COPD, severe asthma) restricting involvement. The sample size was determined using the concept of data saturation, operationally defined as the point at which no new themes or insights emerged from participant interviews [28]. Saturation was reached after 18 patient interviews, and this finding was subsequently verified through two supplementary interviews that yielded no additional data. Participants were coded sequentially as N1 to N20 according to interview order, with demographic details presented in Table 1.

**Table 1:** General information of T2DM patients

ID	Gender	Age (years)	Monthly household income (CNY)	Insurance type	Education level	Residential area	Duration of T2DM	Comorbidity
N1	Male	63	< 5000	Social security	Junior high school	Rural	25 years	DPN
N2	Female	54	5000–10,000	Social security	Primary school	Urban	About 3 years	DPN
N3	Male	57	< 5000	None	Primary school	Urban	About 6–7 years	
N4	Male	71	< 5000	New rural cooperative medical scheme	Primary school	Rural	About 5 months	NA
N5	Male	58	< 5000	Social security	Primary school	Rural	About 4 months	NA
N6	Male	46	< 5000	Social security	High school	Urban	About 3–4 years	NA
N7	Female	50	< 5000	Social security	Primary school	Rural	About 2 years	NA
N8	Female	29	5000–10,000	None	High school	Rural	About 1 years	NA
N9	Female	50	5000–10,000	Social security	High school	Urban	About 7–8 years	DPN
N10	Female	32	< 5000	Social security	Associate degree	Urban	About 6–7 years	NA
N11	Male	57	< 5000	New rural cooperative medical scheme	Primary school	Rural	About 6–7 years	NA
N12	Male	53	> 10,000	Social security	High school	Rural	About 4 months	NA
N13	Male	52	> 10,000	Social security	Associate degree	Urban	About 3 years	DPN
N14	Male	68	< 5000	Social security	Primary school	Urban	About 5 years	NA
N15	Male	50	> 10,000	Social security	Junior high school	Rural	About 2 years	NA
N16	Female	57	> 10,000	Social security	Associate degree	Urban	About 1 years	
N17	Female	72	< 5000	New rural cooperative medical scheme	Primary school	Rural	About20 years	DPN
N18	Male	60	< 5000	Social security	High school	Urban	First diagnosis	NA
N19	Male	55	< 5000	Social security	Associate degree	Rural	About 1 years	NA
N20	Male	54	5000–10,000	Social security	Bachelor degree	Urban	About 7 years	DPN

CNY: China Yuan; DPN: Diabetic Peripheral Neuropathy; DR: Diabetic Retinopathy; NA: Not Applicable

### Data collection

The interview outline was initially developed based on a literature review [19, 29, 30] and discussions among the research team members. Pilot interviews were conducted to optimize the introduction of the questions, making them more natural and understandable, as well as improving the logical flow between the questions to ensure they were pertinent and reasonable. The interview guide is shown in Table 2.

**Table 2:** Interview outline

Main question
1.What’s different about your life since the diabetes diagnosis?
2.Have you heard of tele-nursing services/How would you describe your understanding of tele-nursing services?
3.Would you be willing to use tele-nursing services? Why or why not?
4.What specific needs would you have for tele-nursing services?
5.What changes do you think diabetes tele-nursing will bring?
6.What factors might affect your access to tele-nursing services?
7.What are your thoughts on the future of tele-nursing services?

### Process control

Before the interviews began, the researchers provided participants with a detailed explanation of the study’s objectives, assuring them that all information would remain confidential and be used solely for research purposes. To further reduce social desirability bias, participants were informed that their responses would remain anonymous and that they could withdraw from the study at any time without negative consequences. With extensive experience in clinical diabetes care, the researchers had established strong relationships with the participants, enabling them to recruit eligible patients—those meeting the study’s inclusion and exclusion criteria—from the hospital’s outpatient clinic and inpatient wards. These settings were chosen for their designated quiet rooms, ensuring a private and comfortable environment for the interviews.

Informed consent was obtained from all participants, and the interviews were audio-recorded. Demographic data were also collected. To foster a comfortable and open environment, the interviews were conducted in private rooms, free from distractions. The interviews were carried out by JZ (male) and XXF (female), both with over 10 years of clinical experience. JZ holds a Master’s degree in Nursing and is pursuing a PhD in Nursing, with specialized training in qualitative research methods. ZTL and other co-investigators supervised the interviews, focusing on nonverbal cues such as facial expressions and body language. Their professional backgrounds and training in qualitative research methods contributed to the effective conduct of the interviews.

As clinical nurses, the researchers worked to establish rapport and create a supportive atmosphere, encouraging participants to share their perspectives openly. They listened actively, provided timely feedback, and carefully recorded participants’ tone, emotions, and expressions. To ensure neutrality and minimize social desirability bias, the interviewers remained impartial throughout, avoiding any leading questions or non-verbal cues that could influence responses. Despite the structured format, challenges arose: for example, one participant declined to be recorded, and the researcher took notes to capture key insights instead. After each interview, the researcher promptly organized the data to ensure no information was lost. Given the varying education levels of the participants, no time limit was set for the interviews, which continued until no new insights were offered. Typically, each in-depth interview lasted between 30 and 60 min.

### Data analysis

To conduct the data analysis, we adopted a reflexive thematic analysis following the guidelines outlined by Braun and Clarkee [24]. While two researchers (JZ and ZTL) were primarily responsible for the analysis, all authors actively participated in the process, engaging in discussions about codes and themes.

In Step 1, all authors reviewed the transcripts to familiarize themselves with the content. In Step 2, the first and last authors independently performed line-by-line coding of the interviews. Using a Word document, the transcripts were placed in the left-hand column, the codes in the middle, and any comments or discrepancies in the right-hand column. As they coded, they reflected on emerging patterns and themes across the dataset. If initial codes could not be categorized, the research team discussed and determined their appropriate placement.

In Step 3, after individual coding, the first and last authors reevaluated the codes for consistency and precision. In Step 4, all authors convened to discuss and refine the preliminary themes, focusing on how to structure each theme to ensure both coherence and comprehensiveness. The first and last authors further refined the data, codes, and preliminary themes.

The results of this iterative process were shared with the other authors, leading to Step 5, where the final themes were defined and named. Throughout the process, the methodology encouraged multiple coders to explore diverse interpretations of the data, following a flexible and iterative coding procedure. A detailed illustration of these analytical steps is provided in Table 3. Finally, in Step 6, the results of the analysis are presented in this article, with an example of the analysis process shown in Table 3.

**Table 3:** Example of the analysis process

Themes	Preliminary themes	Codes	Transcript
Insufficient awareness and willingness to use	Low awareness rate	Just heard about tele-nursing	It seems that I’ve heard of it. Is it the nursing service based on the Internet? (N4). Our community has promoted remote nursing, but I haven’t got a specific understanding of it yet (N16, N18).
		Unaware of what it is	I haven’t heard of tele- nursing (except N4, N16 and N18, all of them).
	Low willingness to use	Convenience	It (tele-nursing) seems convenient (N8, N13). It has potential, especially if it can address the specific needs of rural patients and is affordable (N12). It has great potential, especially in urban areas where people are busy (N15).
		Effectiveness to be verified/cost concerns	It has potential, but it’s hard to say if it would be practical (N2). I would need to consider it. If it’s expensive, I can’t afford it (N7). It sounds promising but needs practical implementation to address real needs and concerns (N10). It has great potential if implemented effectively and with attention to patient needs (N12). It sounds useful, but cost is a concern for us (N14).
		Elderly adults’ challenges using smart devices	Many elderly people struggle with using smartphone (N3). Elderly people may find it difficult to use smartphones (N5, N9, N10, N11)

### Quality control

Prior to the interviews, pilot interviews were conducted with colleagues to gather feedback and improve interview skills, ensuring sufficient preparation. During the analysis, researchers set aside preconceived notions to ensure that the themes were tightly connected with the data. All data were given equal importance during coding, and themes were continuously compared with the original data to verify their relevance. Moreover, the audio recordings of the interviews conducted for this study are securely stored and managed by the research team, under the oversight of the institutional ethics committee. Access to the recordings is restricted to the principal investigator and authorized team members only, ensuring that data is handled in compliance with ethical standards and privacy regulations.

## Results

### Participants

Among the 20 T2DM patients, 13 were male and 7 were female, aged 29 to 72 years, with an average age of (54.90 ± 10.95) years. Educational levels included 8 with primary education, 2 with junior high education, 5 with high school education, 4 with associate degree education, and 1 with a bachelor’s degree. Ten patients resided in urban areas, and ten in rural areas.Their characteristics are represented in Table 1.

### Theme

In this study, we developed four key themes: (1) Insufficient Awareness and Willingness to Use, which includes subthemes of insufficient awareness of tele-nursing services among T2DM patients and a low willingness to adopt these services; (2) Needs of Tele-Nursing, encompassing the need for health education, regular follow-ups, and qualified nursing personnel; (3) Facilitators of Tele-Nursing, which includes government policy support, timely diagnosis and convenience, favorable economic conditions, and urban residential location; and (4) Barriers to Tele-Nursing, comprising cost, family economic burden, age, network security concerns, insufficient healthcare resources, and rural residential location. Table 4 presents the themes and their corresponding subthemes. Each theme and subtheme is discussed in the following section, accompanied by illustrative quotations. For privacy, participant names have been replaced with participant numbers (e.g., N1).

**Table 4:** Themes and Sub-themes

Theme	Sub-theme	Explanation/participant experience analysis
Awareness and willingness to use tele-nursing	Insufficient awareness of tele-nursing	Most T2DM patients lacked awareness of tele-nursing services, highlighting the need for better promotion and education by healthcare institutions.
	Low willingness to use tele-nursing	Despite some awareness, patients expressed hesitation due to doubts about the effectiveness and cost, particularly those accustomed to traditional healthcare models.
Needs of tele-nursing	Health education and guidance	Many patients expressed a need for professional guidance on diet, exercise, and medication, revealing gaps in their knowledge of self-management.
	Regular follow-ups	Patients believed regular follow-ups would help with long-term disease management, especially in monitoring post-discharge recovery and complications.
	Qualified nursing personnel	Patients emphasized the importance of nurses with specialized knowledge of diabetes, although some valued nurses’ care and patience over qualifications.
Facilitators of tele-nursing	Government policy support	Participants highlighted that government-backed financial support, such as insurance reimbursement, would encourage them to adopt tele-nursing services.
	Convenience and timely assistant decision	Tele-nursing services were appreciated for their convenience, particularly in reducing hospital visits for minor issues and providing timely intervention, especially for elderly or mobility-impaired patients.
	Economic support	Economic stability, such as sufficient financial resources or adequate insurance coverage, was seen as a major facilitator for adopting tele-nursing services, reducing concerns about affordability.
	Urban infrastructure advantage	Participants in urban areas were more willing to use tele-nursing due to better healthcare infrastructure, reliable internet connectivity, and more resources available compared to rural areas.
Barriers to tele-nursing	Economic burden	Cost concerns were prevalent among lower-income participants, particularly in rural areas, where the additional expense of tele-nursing services was viewed as a significant barrier to its adoption.
	Technological barriers for older adults	Older participants reported difficulties using digital devices required for tele-nursing services, citing lack of familiarity and skills with smartphones and computers.
	Network security	Participants expressed fears about the security of personal information and potential online fraud, which made them hesitant to trust tele-nursing services.
	Healthcare resource limitations	Participants in rural areas raised concerns about the lack of healthcare professionals, particularly nurses, and how this shortage could limit the implementation of tele-nursing services.
	Rural infrastructure challenges	Rural participants identified poor internet connectivity and limited access to healthcare resources as major obstacles to using tele-nursing services in their communities.

### Theme 1: Awareness and willingness to use tele-nursing

#### Insufficient awareness of tele-nursing

The majority of participants indicated that they were not familiar with tele-nursing services. Out of the 20 T2DM patients interviewed, 18 had never heard of the service prior to the interview. For example, N1 stated: “I’ve never heard of this service,” while N4 mentioned: “I think I’ve heard about it in some form, like online consultations, but I don’t really understand it.” Similarly, N16 remarked: “Our community sometimes promotes this, but I haven’t really understood it.”

Only three participants had some level of awareness about tele-nursing services, but their understanding remained superficial. As follows: “It seems that I’ve heard of it. Is it the nursing service based on the Internet?” (N4). “Our community has promoted remote nursing, but I haven’t got a specific understanding of it yet” (N16, N18).

In addition, some interviewees also believe that remote nursing has great potential, but its effectiveness remains to be further verified. Some participants noted: “It has potential, but it’s hard to say if it would be practical” (N2). “It has great potential if implemented effectively and with attention to patient needs” (N12). Other participants also said that there are comparative advantages in urban areas. “It has great potential, especially in urban areas where people are busy” (N15). All in all, this lack of familiarity was widespread across participants regardless of their demographic backgrounds, indicating that tele-nursing had not yet been widely promoted or adequately explained to patients in the sample.

#### Low willingness to use tele-nursing

Even after receiving an explanation of tele-nursing services during the interviews, many participants expressed hesitation or reluctance to use such services. Concerns about the reliability and cost of tele-nursing were prominent among the reasons for their hesitation. Most interviewees stated: “hospitals are still the best option” (N2). Similarly, “This service doesn’t sound free, so I would need to think about it before deciding” (N10).

In addition to concerns about cost and reliability, some participants questioned the practicality of using tele-nursing to replace traditional healthcare services. As N14 remarked: “If everything could be solved online, why do we still need to go to work?” Other participants shared similar sentiments, expressing doubts about whether tele-nursing could fully meet their healthcare needs. As N10 noted: “It sounds promising but needs practical implementation to address real needs and concerns”.

### Theme 2: Needs of tele-nursing

#### Health education and guidance

Most participants expressed a strong need for health education, particularly in areas related to diet, exercise, and medication management. Many felt that they lacked sufficient knowledge about how to properly manage their diabetes on a daily basis. For example, N6 said: “I need regular guidance, like how to eat and exercise”. And, “I take medication and watch my diet. There are no significant inconveniences” (N5).

Similarly, N16 remarked: “I think knowledge dissemination is very important. It’s necessary to strengthen our understanding of this disease”. This reflects a widespread demand for professional guidance to help patients improve their self-management practices. N18 also commented: “In terms of diet, which foods are beneficial for diabetics? Many people are not clear about this”.

Some participants emphasized the importance of receiving consistent and reliable information about diabetes care. As N13 said: “Regular check-ups and dietary advice would be helpful”. They expressed a desire for clear, tailored health education that would help them better understand how to manage their condition, especially diet management. Some of them described: “Diet is a concern since I’m always thinking about what I can or can’t eat” (N10), “I find it tiring to constantly monitor my diet” (N17). And “Tele-nursing should provide diabetes patients with comprehensive guidance, including diet and exercise, and regular blood glucose monitoring” (N18/20).

#### Regular follow-ups

Participants also emphasized the importance of regular follow-ups to ensure effective disease management. Many viewed follow-ups as essential for long-term care, particularly in monitoring potential complications such as diabetic foot and eye conditions. N4 stated: “Just general regular follow-ups”. Similarly, N10 noted: “It would be great if I could have regular follow-ups for my post-discharge recovery”. Several participants expressed concerns about the long-term nature of diabetes management and the need for ongoing professional support. One participant mentioned: “Doctors/nurses could perform preliminary check-ups at your home, via tele-nursing” (N2). And N20 explained: “For issues like diabetic foot and eye screenings, regular check-ups can be arranged”. This indicates a significant demand for follow-ups that focus on preventing complications and managing diabetes-related risks.

#### Qualified nursing personnel

The participants also underscored the need for nursing personnel with specialized knowledge in diabetes care. Many emphasized the importance of receiving care from nurses who understood the complexities of managing T2DM. They said: “Nurses must be qualified and experienced professionals” (N6, N16). And N10 commented: “The service must be related to this department. If you randomly pick someone from another department, they might not know much about diabetes care”. However, some participants prioritized the quality of care and compassion over formal qualifications. For instance, “As long as they genuinely care for the patients, it’s fine. They don’t need to be highly skilled, just genuinely kind to us” (N13). And another noted: “Experienced and caring professionals” (N7).

### Theme 3: Facilitators of tele-nursing

#### Government policy support

Participants frequently mentioned that government policy support, particularly through insurance reimbursement, was a crucial factor in encouraging the use of tele-nursing services. Many participants highlighted that, without government-backed financial support, the cost of these services would remain a significant barrier. As one participant stated: “If the government reimburses it, more people might be willing to use it” (N2). Another participant reflected similar concerns, noting: “If there’s no insurance, it will be too expensive to afford regularly” (N7) and “It would be much better if there is support from the government and medical insurance can be reimbursed” (N20). These responses indicate that the integration of tele-nursing into existing healthcare insurance programs would greatly increase the accessibility and affordability of these services.

#### Convenience and timely assistance

The convenience offered by tele-nursing was frequently cited as a major advantage by participants, particularly among elderly individuals and those with mobility issues. Many appreciated the ability to access healthcare without the need for frequent hospital visits. One participant mentioned: “It’s very convenient; I don’t have to go to the hospital every time” (N5), while another added: “For someone like me, who can’t walk well, this would save me a lot of trouble” (N12). And “Increase the publicity so patients can see the real, tangible conveniences” (N18). In addition, several participants highlighted the potential of tele-nursing for timely assistance, which could help in the early detection of health issues. One participant shared: “I could have caught the issue earlier if I had this service” (N4), emphasizing the importance of early intervention in preventing complications.

#### Economic support

Economic support, whether through personal financial stability or insurance coverage, was seen as an important facilitator for adopting tele-nursing. Participants who had stable financial conditions or access to adequate insurance coverage felt that tele-nursing could be a cost-effective way to manage their health. As one participant remarked: “If insurance helps, I think we can manage the cost” (N3). Another added: “It’s cheaper than frequent hospital visits, especially when insurance covers most of it” (N9). And, “It sounds useful, but cost is a concern for us” (N7, N14). This demonstrates that economic support, particularly in the form of insurance coverage, played a key role in reducing participants’ financial concerns about adopting tele-nursing services.

#### Urban infrastructure advantage

Participants living in urban areas identified the infrastructure advantages of cities, such as reliable internet connectivity and better access to healthcare services, as key facilitators for tele-nursing adoption. One participant mentioned: “We have better networks here, so I think tele-nursing would work well” (N1). Another participant explained: “In the city, you can easily access the internet and have better doctors and nurses. I think it would be more useful here” (N11). In contrast, rural participants pointed out that poor infrastructure and unreliable internet in their areas could pose challenges to tele-nursing implementation.

### Theme 4: Barriers to tele-nursing

#### Economic burden

Many participants, especially those from rural areas or lower-income households, expressed concerns about the economic burden associated with tele-nursing services. They worried that without sufficient financial support, tele-nursing would add to their existing healthcare expenses. One participant shared: “For us, the cost of tele-nursing would just add to our existing hospital bills. We can’t afford that” (N8). Another participant noted: “Even with insurance, the out-of-pocket costs can still be too much” (N5). This highlights the financial strain many participants faced, particularly in managing chronic conditions like diabetes.

#### Technological barriers for older adults

Older participants frequently cited technological barriers as a major obstacle to using tele-nursing services. Many expressed difficulties in using smartphones, computers, or other digital devices required to access tele-nursing platforms. Some participants noted: “Many elderly people struggle with using smartphones” (N3). “Elderly people may find it difficult to use smartphones” (N5, N9, N10, N11). Similarly, “I don’t know how to use these new phones, my children have to help me with everything” (N16). And “I’ve never even touched a computer. How can I use a service like this on my own?” (N19). These technological challenges made many older adults feel unsure about their ability to use tele-nursing services effectively without additional support. In addition, some interviewees said that as elderly people living alone, they had to deal with many things. With no one to help, using tele-nursing was just too hard for them. As two patients noted: “Living alone, I overlook many aspects” (N3). “My wife has passed away, and my children are not around. It’s challenging to manage everything alone” (N13).

#### Network security

Concerns about network security and the safety of personal medical information were also significant barriers to tele-nursing adoption. Many participants expressed fears about potential online fraud and data breaches. One participant said: “You hear so much about online scams these days. I’m worried about sharing my medical information over the internet” (N2). Another participant added: “I don’t trust online systems with my personal information. What if my data gets stolen?” (N14). These concerns were especially common among older participants, who were less familiar with digital platforms and more skeptical about the safety of online healthcare systems.

#### Healthcare resource limitations

Participants from rural areas frequently mentioned the lack of healthcare resources, particularly nurses, as a barrier to tele-nursing. One participant stated: “We barely have enough nurses to cover the basics. How can they handle tele-nursing on top of everything else?” (N20). Another participant expressed similar concerns, noting: “There’s already a shortage of healthcare workers here. I don’t see how they could manage tele-nursing with so few people” (N18). This highlights the pressure on already overworked healthcare professionals in rural regions and the challenge of integrating tele-nursing into these areas.

#### Rural infrastructure challenges

Participants from rural areas also identified poor infrastructure as a significant barrier to tele-nursing. Many mentioned that unreliable internet connectivity and limited access to technology would make tele-nursing difficult to implement. As one participant explained: “We don’t even have reliable internet here. How could we possibly use tele-nursing?” (N5). Another participant noted: “In rural areas like ours, even getting a signal on our phones is hard. There’s no way tele-nursing would work without proper infrastructure” (N9). This demonstrates the infrastructural challenges that many rural participants faced in accessing tele-nursing services.

## Discussion

This study offers valuable insights into the awareness, needs, and barriers regarding tele-nursing among patients with T2DM in China. The thematic analysis identified four key themes: low awareness and willingness to adopt tele-nursing, a strong demand for health education and remote monitoring, facilitators like government support, and barriers such as technological challenges and rural healthcare limitations. Although patients demonstrated limited familiarity with tele-nursing, they expressed a clear need for services that support self-management and regular follow-ups. Despite technological barriers, particularly in rural areas, tele-nursing was perceived as a cost-effective solution with the potential to improve diabetes management, provided that infrastructure and digital literacy issues are addressed. Our findings suggest that increasing patients’ awareness of tele-nursing is essential for its successful implementation.

The study revealed that most patients had little to no prior knowledge of tele-nursing, with only three participants having some understanding. Interviews highlighted that limited access to information, particularly in rural areas, contributed to this lack of awareness. In addition, some participants expressed reluctance to use smart devices, citing their reluctance to use smart devices as a reason for refusing tele-nursing services. In fact, older adults often face challenges in accepting and utilizing tele-nursing [31]. Notwithstanding, targeted technical training programs and family education initiatives have proven to be effective strategies for enhancing elderly patients’ technical skills and strengthening family support systems for tele-nursing [32]. Improving digital literacy among senior populations can significantly boost telehealth adoption, bridging the digital divide in healthcare delivery.

This study demonstrates that, targeted training and awareness campaigns—focusing on the benefits of tele-nursing—can increase acceptance and awareness of tele-nursing services [33]. Odnoletkova et al. [34] found that nurse-led tele-nursing significantly reduced HbA1c levels and improved disease management knowledge among T2DM patients. Additionally, many participants believed that tele-nursing would be easier to implement in urban areas than in rural regions, likely due to a lack of understanding of its advantages in rural settings. Telemedicine and tele-nursing have the potential to overcome time and spatial limitations, making them particularly beneficial in areas with underdeveloped medical infrastructure [35].

According to these results, the very first phase in the implementation process should be to increase the promotion of tele-nursing.

Although most participants demonstrated limited awareness of tele-nursing, patients expressed a desire for a range of health management services, such as blood sugar monitoring, food education, and exercise guidance, after the researcher introduced them to tele-nursing. Other needs include medication reminders, screening for diabetic complications, and regular follow-ups. While these findings point to a need for support in diabetes self-management, they do not indicate a universal inadequacy in knowledge and skills, but rather highlight areas where additional guidance could be beneficial. Tele-nursing can meet these demands by providing real-time support, consistent with some findings in the literature [36, 37]. Additionally, the tele-nursing needs of the interviewees focus on specialized diabetes care, distinct from general basic care (e.g., home injections, infusions, dressing changes). Research indicates that tele-nursing, supported by multidisciplinary teams, can achieve professional interventions, such as enhancing lifestyle interventions through virtual technology to help control clinical symptoms in T2DM patients [38, 39]. To summarize, patients anticipate receiving specific tele-nursing, such as diet guidance, exercise, blood glucose control, prompt information support, and decision-making for daily management.

In the first place, patients highlighted the importance of cost considerations and insurance coverage when evaluating tele-nursing services. Establishing a standardized and systematic nursing service process to balance treatment costs is critical for diabetes self-management [40]. If tele-nursing becomes more widespread, incorporating it into medical insurance systems could reduce the financial burden on patients [41]. Therefore, the design of tele-nursing platforms should prioritize user-friendliness, security, and efficiency.As shown, the main factor influencing T2DM patients’ decision to adopt telemedicine is its cost. In the future, policymakers should focus on creating reasonable pricing structures and supporting policies to alleviate the economic burden on patients.

In the second place, some respondents mentioned concerns about the availability of healthcare resources in primary care, such as nurse-patient ratios and service reliability. However, most respondents suggested outdated medical equipment or insufficient nursing staff as the main reasons for preferring tertiary hospitals. Although primary healthcare institutions are essential in managing long - term conditions effectively, most respondents still preferred tertiary hospitals due to outdated medical equipment or insufficient nursing staff [42, 43]. Yao et al. [44] found that a weak health service system is a major obstacle in diabetes management. Inadequate service capacity of community medical units, lack of specialized disease care teams, and poor healthcare environments result in low willingness of T2DM patients to seek primary care [45]. The above indicates that telecare largely depends on large hospitals. Additionally, respondents expressed a need for qualified personnel in tele-nursing to ensure the quality of care. Tele-nursing staff should be professionally certified to guarantee care quality [41]. Health authorities should also enhance the training and management of telehealth service personnel [46]. Related studies indicate that tertiary hospitals can use tele-technology to distribute quality medical resources to primary care, strengthening primary healthcare or directly serving community patients, thereby compensating for the lack of primary healthcare resources and ultimately benefiting patients [47, 48]. The findings advocate for policy initiatives that facilitate knowledge transfer from specialized diabetes care units to primary health institutions through digital nursing platforms, creating sustainable patient-centered care models.

As mentioned in the discussion on willingness to use, some elderly respondents expressed concerns about their ability to use smart devices, particularly regarding the complexity of operations and potential information security risks. Lastly, a few patients indicated that privacy and security issues were significant concerns that could deter them from using online services. While these elderly T2DM patients may require technical support to use telemedicine, some demonstrated interest in telehealth interventions [49]. Although technical barriers exist, they are not insurmountable obstacles to tele-nursing adoption. On the other hand, targeted social support training, simplifying the user interface of smart devices, and improving digital literacy can help alleviate these technology-related barriers to the use of tele-nursing [50].

In summary, current findings revealed limited awareness and low adoption intent toward tele-nursing among interviewed patients with T2DM. It is important to acknowledge that China’s tele-nursing and telemedicine fields encounter difficulties with inequitable distribution of resources, implementation readiness, working conditions, and evidence foundation. Therefore, a predominant preference for in-person consultations at large hospitals persists, underscoring a critical gap in healthcare service delivery. The research team identified insufficient understanding of tele-nursing benefits as the primary barrier to utilization. To address this, targeted educational campaigns emphasizing tele-nursing advantages—such as reduced travel burdens and continuous glycemic monitoring—are urgently needed. Furthermore, participants expressed unmet needs for accessible diabetes self-management education, a gap that tele-nursing platforms could strategically fill through structured knowledge dissemination and remote skill-building interventions.

### Methodological considerations

This study, based on 20 in-depth interviews, offers valuable insights into the needs and influencing factors of tele-nursing services for patients with T2DM. However, due to the small sample size, the generalizability of the findings may be limited. According to Malterud [51], when qualitative data are rich and insightful, a smaller sample size can still be sufficient. While the interviews in this study provided comprehensive data, the small sample size may not fully reflect the diverse needs across different regions and patient groups. What’s more, the researchers’ prior clinical experience and preconceived notions may have subtly shaped the data analysis and interpretation. Given that the primary interviewers were experienced clinical nurses, this helped ensure the interviews stayed focused on the research objectives. However, their clinical background may have led to an overemphasis on patients’ technological challenges and financial concerns, potentially overlooking other important social and psychological factors. To mitigate this bias, the study implemented strategies to enhance the objectivity of the analysis. All researchers actively participated in the data collection, coding, and theme development, engaging in reflective analysis to maintain transparency throughout the process. The research team also held regular discussions to critically examine the potential influence of personal biases on the interpretation of the data. Despite these efforts, the clinical experience of the researchers may have still influenced the interpretation of the findings to some extent.

### Future research

Future research will primarily focus on deepening the understanding and practice of tele-nursing for patients with T2DM. Firstly, the research will combine existing tele-nursing evidence with advanced technologies to develop a more universal tele-nursing platform, and explore the integration with artificial intelligence to create intelligent tele-nursing platforms. Through the gradual implementation of pilot projects, patients will experience the convenience and benefits brought by tele-nursing, while also enhancing their awareness and engagement with tele-nursing. Secondly, the combination of quantitative and qualitative research evidence will strengthen the generalizability of the study results. At the policy level, it is necessary to establish an economic evaluation model to quantitatively compare the differences in healthcare expenditures, productivity losses, and other aspects between tele-nursing and traditional outpatient care, providing strong evidence for reforming healthcare insurance payment systems [52]. Finally, integrating multidisciplinary teams into tele-nursing models can enhance care for T2DM patients. Combining expertise from dietitians, diabetes educators, and mental health professionals can create a holistic approach tailored to patients’ complex needs, offering valuable guidelines for future practice.

## Conclusion

In conclusion, this study identified low awareness of tele-nursing among T2DM patients as a key issue to be addressed in tele-nursing implementation. Patients expect to receive such specialized tele-nursing services as health education, glucose monitoring, dietary guidance, and regular follow-up visits. The findings also highlight core tele-nursing factors, including policy support and digital accessibility, as well as barriers such as technological challenges, economic issues, and urban-rural disparities.

## Data Availability

Data is not provided within the manuscript. However, it can be obtained from the corresponding author for legitimate scientific research purposes.

## Abbreviations

T2DM: Type 2 Diabetes Mellitus
HbA1c: Hemoglobin A1c
CNY: China Yuan
COREQ: Consolidated Criteria for Reporting Qualitative Research
DPN: Diabetic Peripheral Neuropathy
DR: Diabetic Retinopathy
NA: Not Applicable
